# Jackson County Medical Society—First Regular Meeting

**Published:** 1867-08

**Authors:** O. B. Ormsby

**Affiliations:** Secretary


### JACKSON COUNTY MEDICAL SOCIETY—FIRST REGULAR MEETING.

The Jackson County Medical Society met pursuant to adjournment of the primary meeting in Carbondale, Ill., July 3d, at 10 o'clock A.M.

Dr. James Roberts, in assuming the Chair, remarked that he was much gratified by the honor conferred upon him in his election as President. He hoped he was only the first of a long line of Presidents which should occupy the Executive Chair of a Society increasing in numbers and wisdom, as its years increase.

The minutes of the primary meeting, including the Constitution and By-Laws, were read.

Upon motion of Dr. Ormsby, Dr. Roberts was requested to present to the Society, at its next regular meeting, an essay on the subject of Milk Sickness, to which request he acceded, and, in so doing, expressed the hope that members of the Society would enhance the value of the paper, by preparation for a full discussion on the subject, from the standpoint of each one. He had been engaged in practice in this vicinity for nearly thirty years, and was well aware that the disease in question merited our attention from the fact that it is not ten or twenty miles distant, but is here, as it were, at our very doors.

Dr. Ormsby rose to explain, that notwithstanding Dr. Roberts was to present a paper to the Society at its next regular meeting, it would be not only proper, but highly desirable, that any other members should present such papers and reports of cases as they deem proper to lay before the Society, and he hoped that no member would allow his native modesty to prevent his being heard from. Modesty does not often kill individuals, but it sometimes takes all the vim out of a Society.

Dr. Roberts called attention to the fact that the editor of the Carbondale *Era* has offered to publish any notices for the Society gratis, and has requested a "resume" of our proceedings for publication. He thought it might be well to furnish a short account of our meeting to the *Era*, as a reminder to our professional brethren that such an organization as the County Medical Society is in existence.

On motion of Mr. Ditzler, the Secretary was instructed to furnish a "resume" of these proceedings, together with such extracts from the Constitution and By-Laws as he may deem proper, to the *Era*, for publication.

On motion of Dr. Ormsby,

*Resolved*, That a copy of these proceedings be sent to the CHICAGO MEDICAL EXAMINER.

After a desultory discussion upon the subject of Medical Education, in which the recent action of the "Convention of Teachers of Medical Colleges," was heartily and unanimously proved, the Society adjourned.

O. B. ORMSBY, M.D., Secretary.

